# Attitudes, Knowledge and Factors Associated with Human Papillomavirus (HPV) Vaccine Uptake in Adolescent Girls and Young Women in Victoria, Australia

**DOI:** 10.1371/journal.pone.0161846

**Published:** 2016-08-26

**Authors:** Iris L. Y. Tung, Dorothy A. Machalek, Suzanne M. Garland

**Affiliations:** 1 Department of Microbiology and Infectious Diseases, The Royal Women’s Hospital, Parkville, Victoria, Australia; 2 Murdoch Childrens Research Institute, Parkville, Victoria, Australia; 3 Department of Obstetrics and Gynaecology, The University of Melbourne, Victoria, Australia; Universidade Estadual de Maringa, BRAZIL

## Abstract

**Background:**

Human papillomavirus (HPV) vaccination targets high-risk HPV16/18 that cause 70% of all cancers of the cervix. In Australia there is a fully-funded, school-based National HPV Vaccination Program which has achieved vaccine initiation rate of 82% among age-eligible females. Improving HPV vaccination rates is important in the prevention of morbidity and mortality associated with HPV-related disease. This study aimed to identify factors and barriers associated with uptake of the HPV vaccine in the Australian Program.

**Methods:**

Between 2011 and 2014, females aged 18–25 years, living in Victoria, Australia who were offered HPV vaccination between 2007 and 2009 as part of the National HPV Vaccination Program, living in Victoria, Australia were recruited into a a young women’s study examining effectiveness of the Australian National HPV Vaccination Program. Overall, 668 participants completed the recruitment survey, which collected data of participants’ demographics and HPV knowledge. In 2015 these participants were invited to complete an additional supplementary survey on parental demographics and attitudes towards vaccinations.

**Results:**

In 2015, 417 participants completed the supplementary survey (62% response rate). Overall, 19% of participants were unvaccinated. In multivariate analyses, HPV vaccination was significantly associated with their being born in Australia (p<0.001), having completed childhood vaccinations (p<0.001) and their parents being main decision-makers for participants’ HPV vaccination (p<0.001). The main reason reported for HPV non-vaccination was parental concern about vaccine safety (43%). Compared with HPV-vaccinated participants, those unvaccinated were significantly more likely to be opposed to all vaccines, including HPV vaccines (p<0.001) and were less likely to consider vaccinating their own children with all vaccines (p = 0.033), including HPV vaccines (p<0.001). Overall, 61% of unvaccinated participants reported that a recommendation from GPs would increase HPV vaccine acceptance.

**Conclusions:**

Attitudes towards general health, vaccinations in general, as well as HPV vaccines are important in HPV vaccine uptake. Long-term monitoring of the knowledge, attitude and beliefs towards HPV vaccination in the community is critical to ensure a continued high uptake of the vaccine and success of the program.

## Introduction

Persistent infection with high-risk human papillomavirus (HPV) types HPV16 and HPV18 cause 70% of all cancers of the cervix worldwide, and a portion of cancers of the vagina, vulva, anus, penis and head and neck [1–4]. In 2007, the Australian Government introduced a fully-funded National HPV Vaccination Program using a three dose course of the quadrivalent HPV (4vHPV) vaccine (that protects against infection by HPV types 16, 18, 6 and 11). Between 2007 and 2009, all girls aged 12–18 years were offered vaccination through schools, with a catch-up through community provided for women up to the age of 26 years [5–7]. In 2013, the program was extended to include 12–13 year old boys. Vaccination of girls and boys aged 12–13 is ongoing in schools under the National Immunisation Program [8].

In 2013, data from the National HPV Vaccination Program Register (NHVPR) showed that 86% of Australian adolescent girls, aged 12 to 13 received at least one vaccine dose, with 77% receiving all three doses, whilst 64% of those aged 20 to 26 received at least one dose [9]. High vaccine coverage has also been achieved in some other programmes internationally. For example the United Kingdom (school-based delivery) [10] and Denmark (clinic based) report 91% and 89% of school-aged girls have received at least one dose of the vaccine, with 87% and 74% receiving all three doses, respectively [11, 12]. Contrastingly, in the US, where HPV vaccination delivery is through healthcare providers on a reimbursement system, substantially lower vaccination rates have been achieved. In 2013, 57% of school-aged females in the US had received at least one dose of the vaccine and 38% all three doses [13].

Published research exploring attitudes, knowledge and socio-demographic factors associated with uptake of the HPV vaccine in high-income countries are mostly US-based and yield inconsistent findings [14–18]. In Australia, prior surveys have identified a number of socio-demographic, lifestyle and behavioural factors associated with vaccine uptake in the catch up phase but all have focused on individual factors [19, 20]. Few studies have explored reasons for non-vaccination with respect to parental factors. Given that parental views and attitude are likely to shape those of the child [21] and parental consent for HPV vaccination is required for girls under the age of 18, understanding parental factors towards the HPV vaccine is crucial to maintain high vaccine uptake. This study aimed to investigate individual and parental factors associated with uptake of the HPV vaccine among young women in the 2007 to 2009 catch-up phase of the National HPV Vaccination Program.

## Methods

### Recruitment

**Fig 1:**
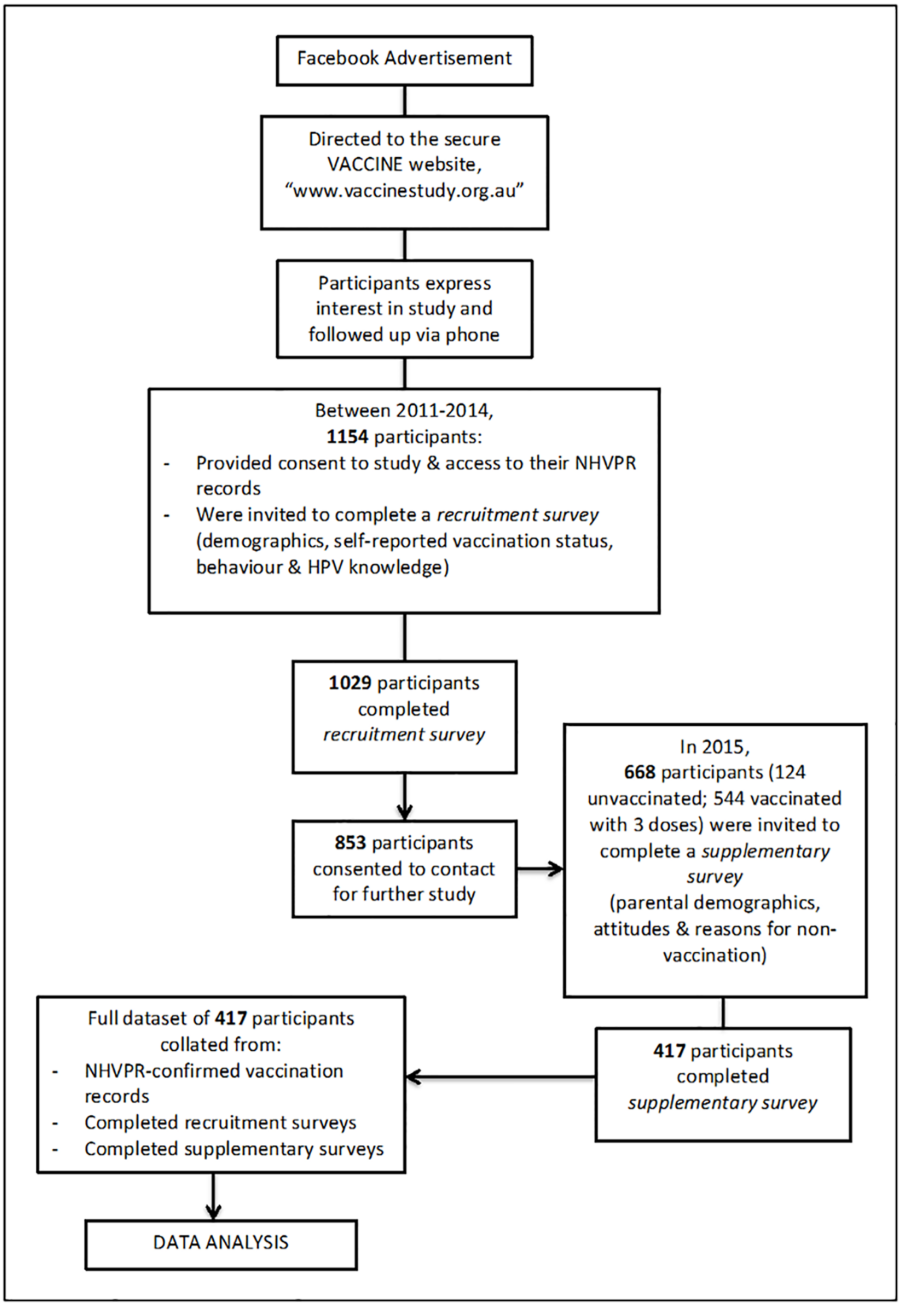
Flowchart outlining study design and recruitment.

Between September 2011 and December 2014 women aged 18–25 years, living in Victoria, Australia were recruited into the Vaccine Against Cervical Cancer Impact and Effectiveness (VACCINE) Study, through targeted advertisements placed on the social media website Facebook (Fig 1) as previously described [22]. Briefly, Facebook users who clicked on the VACCINE advertisement were taken to a study website, which provided details about the study and offered the opportunity to lodge an expression of interest in participating via a secure online form. Participants were then contacted by telephone by the study investigators and provided with an opportunity for questions and verbal consent. Those who provided verbal consent were sent an email with a link to the online participant information and consent form, which was hosted on the secure website SurveyMonkey.com. As part of the written consent, participants were asked to permit the researchers to verify their self-reported HPV vaccination details with the National HPV Vaccination Program Register (NHVPR). The NHVPR was established to monitor HPV vaccine uptake across Australia [23] and collects data on date of HPV vaccination, number of doses received and location of dose administration. Once informed consent was provided, a unique study number was issued, a further email was sent containing a link to the online questionnaire and a study pack containing materials for vaginal sample self-collection was mailed to the participant’s nominated address. This study was approved by The Royal Women’s Hospital Human Research and Ethics Committees (HREC number 11/15).

For the current study, VACCINE participants who agreed to be contacted for additional research and who were confirmed as being vaccinated or unvaccinated by the NHVPR were invited to complete a supplementary survey (n = 1154). Participants were categorised as being “vaccinated” if they had received three doses of the vaccine, as recorded in the NHVPR. Participants were categorized as being “unvaccinated” if they self-reported not receiving the vaccine and the NHVPR had no record of any HPV vaccine dose being delivered. Those with fewer than three doses were excluded from the supplementary survey. Participants who self-reported that they had received the HPV vaccine overseas and therefore did not have a NHVPR record were excluded from the study.

### Measures

Responses to two web-based surveys, including the original 2011–2014 VACCINE study survey and a supplementary survey administered in this study were collected. The former collected information on participants’ demographics, sexual history, cervical screening history and HPV knowledge [24], while the latter collected information about participants’ health, childhood vaccination history, attitudes towards vaccination, reasons for non-vaccination and parental characteristics including parental country of birth. Attitudes were assessed using seven-point Likert scales.

### Statistical analyses

Univariate and adjusted logistic regression analysis were performed with Odds Ratios (ORs) and 95% confidence intervals (CI) generated to identify factors associated with vaccination. Factors included age at program commencement, country of birth, completion of childhood vaccinations, maternal and paternal countries of birth. All variables that were associated with vaccination at p<0.100 in univariate analysis were included in the initial multivariable model. A final multivariable model was obtained by performing backward elimination of statistically non-significant variables, each time assessing for confounding and co-linearity, until only statistically significant variables remained. Descriptive statistics were used to examine reasons for HPV non-vaccination in unvaccinated participants. Childhood socioeconomic status (lower or upper 50th centile classified as more or less disadvantaged) was derived from the Australian Bureau of Statistics Index of Relative Socioeconomic Disadvantage for each individual’s residential postcode. Childhood residential area (major city or) was based on the Accessibility/Remoteness Index of Australia (ARIA) classification[25, 26]. Chi-squared tests were performed to compare differences between HPV-vaccinated and unvaccinated cohorts. Data analyses were performed using STATA version 13 (Stata corporation, College Station, TX, US).

## Results

### Participant recruitment

Between 2011 and 2014 1,154 women who were eligible to receive the free HPV vaccine during the 2007–2009 catch-up period were recruited into the VACCINE study. The women in this study aged 18–25 years in 2011 to 2014 would have been 11–21 years of age at the commencement of the program and therefore had access to the vaccine for free through either the school based or catch up program. Overall, 124 unvaccinated and 544 fully vaccinated participants who consented to be contacted for future studies were invited to the 2014 supplementary survey. Of these, 417 completed the survey (Fig 1). Responders and non-responders for the supplementary survey were not significantly different with respect to age at recruitment (p = 0.378), country of birth (p = 0.832), area of remoteness (p = 0.195), socioeconomic status (p = 0.677) and HPV vaccination status (p = 0.594).

### Cohort characteristics

Overall, 337 (81%) participants were fully HPV-vaccinated and 80 (19%) were unvaccinated. Of the vaccinated participants, 68% received their first vaccine dose at school and 32% at a general practice (GP). The median (interquartile range) age at HPV vaccination for the first dose was 16 years (IQR: 15–18), with no significant difference in age between school- and GP-vaccinated participants (p = 0.262). Table 1 shows the distribution of the population characteristics of the 417 women included in this analysis. The median (interquartile range) age of participants at the supplementary survey was 24 (IQR: 22–25), with no differences by vaccination status. Overall, the majority of women (77%) were under 18 years of age at the commencement of the program. The vast majority were born in Australia (87%), with both parents being Australian born (63%), and 80% resided in metropolitan areas. Just over a quarter (28%) of participants reported that neither of their parents practice any religion.

**Table 1:** Demographic, lifestyle and sexual behaviour characteristics among 417 participants who were offered HPV vaccination between 2007 and 2009 as part of the National HPV Vaccination Program, living in Victoria, by NHVPR confirmed vaccination status.

Characteristic		Total (N = 417)	Unvaccinated (n = 80)	Fully vaccinated (n = 337)	p-value
		n (%)	n (%)	n (%)	
**Birth cohort** ^1^	1996–1994	46 (11.0)	8 (10.0)	38 (11.3)	0.318
	1993–1992	95 (22.8)	21 (26.3)	74 (22.0)	
	1991–1990	124 (29.7)	17 (21.3)	107 (31.8)	
	1989–1988	108 (25.9)	26 (32.5)	82 (24.3)	
	1987–1986	44 (10.7)	8 (10.0)	36 (10.7)	
**Age at program commencement**	11–17 years old	320 (76.7)	59 (73.8)	261 (77.5)	0.482
	18–21 years old	97 (23.3)	21 (26.3)	76 (22.6)	
**Country of Birth**	Australia	361 (87.2)	53 (66.3)	308 (92.2)	**<0.001**
	Other^4^	53 (7.8)	27 (33.8)	26 (7.8)	
**Childhood area of residency**^2^	Major city	239 (66.0)	35 (68.6)	204 (65.6)	0.672
	Regional or remote	123 (34.0)	16 (31.4)	107 (34.4)	
**Childhood SES**^3^	More disadvantaged	135 (37.3)	20 (39.2)	115 (37.0)	0.759
	Less disadvantaged	227 (62.7)	31 (60.8)	196 (63.0)	
**Parental country of birth**	Both Australian born	259 (63.0)	37 (48.1)	222 (66.5)	**<0.001**
	One parent born overseas^5^	80 (19.5)	12 (15.6)	68 (20.4)	
	Both parents born overseas^5^	72 (17.5)	28 (36.4)	44 (13.2)	
**Parental religion**	Both non-religious	113 (27.6)	24 (31.6)	89 (26.7)	0.517
	One parent religious	100 (24.5)	20 (26.3)	80 (24.0)	
	Both parents religious	196 (47.9)	32 (42.1)	164 (49.3)	
**Childhood vaccinations**	Incomplete	23 (5.5)	13 (16.3)	10 (3.0)	**<0.001**
	Complete	394 (94.5)	67 (83.8)	327 (97.0)	
**Main decision maker regarding receiving the HPV vaccine**	Self	181 (43.4)	54 (67.5)	127 (37.7)	**<0.001**
	One or both parents	236 (56.6)	26 (32.5)	210 (62.3)	
**Tobacco use**	Never smoked	336 (81.8)	57 (72.2)	279 (84.0)	**0.014**
	Past or current smoker	75 (18.3)	22 (27.8)	53 (16.0)	
**Private health insurance**	No	174 (41.7)	44 (55.0)	130 (38.6)	**0.007**
	Yes	243 (58.3)	36 (45.0)	207 (61.4)	
**Age at first sex**	11–17	185 (55.9)	30 (50.0)	155 (57.2)	0.310
	18–24	146 (44.1)	30 (50.0)	116 (42.8)	
**Lifetime number of sexual partners**	0	81 (20.0)	17 (22.4)	64 (19.5)	0.530
	1–3	167 (41.2)	27 (35.5)	140 (42.6)	
	4+	157 (38.8)	32 (42.1)	125 (38.0)	
**Current contraceptive use**	Yes	296 (89.2)	49 (81.7)	247 (90.8)	**0.039**
	No	36 (10.8)	11 (18.3)	25 (9.2)	
**Date of last Pap smear**	Within last two years	199 (89.6)	31 (81.6)	168 (91.3)	0.073
	More than two years ago	23 (10.4)	7 (18.4)	16 (8.7)	

^1^: Birth year corresponds to women between 11–21 years old as of the 1^st^ of January 2007 who were offered HPV vaccination between 2007 and 2009 as part of the National HPV Vaccination Program;

^2^: based on the Accessibility/Remoteness Index of Australia (ARIA) classification;

^3^: based on the Australian Bureau of Statistics Index of Relative Socioeconomic Disadvantage for each individuals residential postcode;

^4^: Other countries of birth include New Zealand, China, Fiji, Finland, Germany, Hong Kong, India, Indonesia, Japan, Kenya, Malaysia, Singapore, Serbia, South Africa, Sri Lanka, Sweden, United Kingdom, US, Vietnam;

^5^: Overseas countries correspond to New Zealand, Bangladesh, Brunei, Canada, Chile, China, Egypt, Fiji, Finland, France, Germany, Hong Kong, India, Indonesia, Israel, Italy, Kenya, Latvia, Lebanon, Malaysia, Malta, Mauritius, Netherlands, Pakistan, Papua New Guinea, Philippines, Poland, Republic of Malawai, Sweden, Singapore, Spain, South Africa, Sri Lanka, Tanzania, United Kingdom, US, Vietnam, Zimbabwe.

Abbreviations: SES: Socioeconomic status; Pap: Papanicolaou. Numbers do not always total 417 because of small amounts of missing data.

Compared with unvaccinated women, those HPV-vaccinated were significantly more likely to be born in Australia (<0.001), be non-smokers (p = 0.014) and have private health insurance (p = 0.007). HPV-vaccinated and unvaccinated participants were not different with respect to age at first sex (p = 0.310) and lifetime number of sexual partners (p = 0.530). However, HPV-vaccinated participants were more likely to use contraception (p = 0.039) and have had a Pap smear within the last two years (p = 0.073); however, the latter did not reach significance (Table 1).

### Factors associated with HPV vaccination

The results of univariate and multivariable analyses are presented in Table 2. In univariate analysis, being vaccinated was significantly associated with being born in Australia (p<0.001), having completed childhood vaccinations (p<0.001), having one or both parents being born in Australian (p<0.001) and one or both parents being main decision makers regarding receiving the HPV vaccine (p<0.001). Age, socioeconomic status and area of remoteness during childhood, and parental religion were not significantly associated with being vaccinated. In multivariable analysis, factors that remained significantly associated with HPV vaccination included being born in Australia (p<0.001), having completion of childhood vaccinations (p<0.001) and one or both parents being main decision maker regarding receiving the HPV vaccine (p<0.001). The results were the same when the analysis was limited to only women who were under 18 years of age at commencement of the National HPV Vaccination Program in 2007.

**Table 2:** Factors associated with receipt of the HPV vaccine between 2007 and 2009 as part of the National HPV Vaccination Program among 417 female participants living in Victoria, Australia, overall and stratified by age-group at commencement of the HPV vaccination program.

Factors		Overall cohort (N = 417)				< 18 years old at program commencement (n = 320)		18 years or older at program commencement (n = 97)	
		OR (95% CI)	p–value	Adjusted ^a^ OR (95%CI)	p-value	Adjusted ^b^ OR (95%CI)	p-value	Adjusted ^b^ OR (95%CI)	p-value
**Age at program commencement**	11–17 years old	1.00	0.482	1.00	0.949				
	18–21 years old	0.82 (0.47–1.43)		0.97 (0.42–2.27)					
**Country of birth**	Australia	1.00	**<0.001**	1.00	**<0.001**	1.00	**0.002**	1.00	0.399
	Other	0.17 (0.09–0.31)		0.21 (0.09–0.55)		0.18 (0.06–0.53)		0.38 (0.04–3.89)	
**Childhood SES**^1^	More disadvantaged	1.00	0.759						
	Less disadvantaged	1.10 (0.56–2.02)							
**Childhood area of residency**^2^	Major city	1.00	0.672						
	Regional or remote	1.15 (0.61–2.17)							
**Childhood vaccinations**	Incomplete	1.00	**<0.001**	1.00	**<0.001**	1.00	**<0.001**	1.00	0.130
	Complete	6.34 (2.67–15.07)		6.99 (2.71–17.99)		10.81 (3.2–36.32)		4.00 (0.63–20.71)	
**Parental country of birth**	Both Australia born	1.00		1.00		1.00		1.00	
	One parent born overseas	0.94 (0.47–1.91)	0.874	1.19 (0.55–2.62)	0.651	1.42 (0.56–3.57)	0.457	0.78 (0.17–3.45)	0.694
	Both parents born overseas	0.26 (0.15–0.47)	**<0.001**	0.82 (0.32–2.07)	0.671	0.95 (0.33–2.73)	0.926	0.42 (0.05–3.57)	0.483
**Main decision maker regarding receiving the HPV vaccine**	Self	1.00	**<0.001**	1.00	**<0.001**	1.00	**0.004**	1.00	0.097
	One or both parents	3.43 (2.05–5.76)		3.10 (1.66–5.80)		2.72 (1.37–5.38)		6.21 (0.72–53.50)	
**Parental religion**	Both non-religious	1.00							
	One parent religious	1.08 (0.55–2.10)	0.824						
	Both parents religious	1.38 (0.77–2.49)	0.281						

^1^: Socioeconomic status (SES) based on the Accessibility/Remoteness Index of Australia (ARIA) classification;

^2^: based on the Australian Bureau of Statistics Index of Relative Socioeconomic Disadvantage for each individuals residential postcode;

^a^: Adjusted for age at program commencement, childhood vaccinations, country of birth, parental country of birth, main decision maker regarding HPV vaccination and participant age;

^b^: Adjusted for all the same variables as in (a) except for age at program commencement.

### HPV knowledge, source of information and attitudes towards HPV vaccination

Participants were asked to complete an HPV knowledge test and report their most trusted sources of HPV information. Overall, 97% of participants knew HPV causes cervical cancer and 99% knew regular Pap smears were required after HPV vaccination (Table 3). Two thirds (68%) knew HPV causes genital warts. HPV-vaccinated participants were more likely to correctly identify that HPV vaccination reduces the risk of cervical cancer (p<0.001), that regular Pap smears are required after HPV vaccination (p = 0.015), and that HPV vaccine protects against 70% of cervical cancers (p = 0.027). Overall, 83% of participants reported their GP to be the most trusted source of HPV information. In addition, compared with unvaccinated, vaccinated participants were significantly more likely to report GPs as their most trusted source of HPV information (85.5% versus 71.3%, p = 0.003).

**Table 3:** HPV knowledge among 417 participants who were offered HPV vaccination between 2007 and 2009 as part of the National HPV Vaccination Program, living in Victoria, Australia, stratified by NHVPR confirmed vaccination status. Frequency of correct responses presented.

Knowledge	Total (N = 417)	Vaccinated (N = 337)	Unvaccinated (N = 80)	p-value
	n (%)	n (%)	n (%)	
**HPV infection causes genital warts**	261 (67.6)	221 (69.5)	40 (58.8)	0.088
**HPV infection causes cervical cancer**	374 (96.9)	310 (97.5)	64 (94.1)	0.147
**HPV vaccination reduces risk of cervical cancer**	398 (95.4)	331 (98.2)	67 (83.8)	**<0.001**
**Regular Pap smears are still required after HPV vaccination**	393 (98.7)	323 (99.4)	70 (95.9)	**0.015**
**HPV vaccine protects against 70–80% of cervical cancers**	287 (72.1)	242 (74.5)	45 (61.6)	**0.027**
**Abnormal Pap smears may still occur after HPV vaccination**	370 (93.0)	303 (93.2)	67 (91.8)	0.662

Next, participants were asked to report their agreement with a number of statements about vaccination on a seven-point Likert scale. The results of this are presented in Table 4. Unvaccinated participants were significantly more likely to report neutral to opposing views towards vaccinations in general (p = 0.001) and towards HPV vaccination (p<0.001), than vaccinated participants. In addition, unvaccinated participants were more likely to report neutral or opposing views when asked if they would vaccinate their children with the HPV vaccine now or in the future (p = 0.033). Attitudes around stigma of HPV vaccination and that it implied promiscuity due to HPV being sexually transmitted were not significantly different between the two groups (Table 4).

**Table 4:** Attitude towards vaccination and the HPV vaccine among 417 participants who were offered HPV vaccination between 2007 and 2009 as part of the National HPV Vaccination Program, living in Victoria, Australia, stratified by NHVPR confirmed vaccination status.

		Total (N = 417)	Vaccinated (N = 337)	Unvaccinated (N = 80)	p-value
		n (%)	n (%)	n (%)	
**I am opposed to vaccination in general (any vaccine)**	Disagree	393 (94.5)	324 (96.4)	69 (86.3)	**0.001**
	Neutral	17 (4.1)	8 (2.4)	9 (11.3)	
	Agree	6 (1.4)	4 (1.2)	2 (2.5)	
**I would not vaccinate my children with any vaccine now or in the future**	Disagree	393 (94.5)	322 (95.8)	71 (88.8)	**0.033**
	Neutral	17 (4.1)	11 (3.3)	6 (7.5)	
	Agree	6 (1.4)	3 (0.9)	3 (3.8)	
**I am opposed to HPV vaccination**	Disagree	383 (92.7)	322 (96.7)	61 (76.3)	**<0.001**
	Neutral	25 (6.1)	9 (2.7)	16 (20.0)	
	Agree	5 (1.2)	2 (0.6)	3 (3.8)	
**I would not vaccinate my children with the HPV vaccine now or in the future**	Disagree	378 (91.1)	319 (95.2)	59 (73.8)	**<0.001**
	Neutral	31 (7.5)	13 (3.9)	18 (22.5)	
	Agree	6 (1.5)	3 (0.9)	3 (3.8)	
**There is a stigma attached to the HPV vaccine**	Disagree	296 (71.2)	242 (72.0)	54 (67.5)	0.193
	Neutral	107 (25.8)	86 (25.6)	21 (26.3)	
	Agree	13 (3.1)	8 (2.4)	5 (6.3)	
**Having the HPV vaccine implies a person may be or may become sexually promiscuous**	Disagree	324 (77.9)	268 (79.8)	56 (70.0)	0.083
	Neutral	82 (19.7)	62 (18.5)	20 (25.0)	
	Agree	10 (2.4)	6 (1.8)	4 (5.0)	

Attitudes were measured using a 7-point Likert scales (Strongly disagree and Disagree were grouped as “Disagree”, Neutral, Agree and Strongly Agree were grouped as “Agree”)Numbers do not always total 417 because of small amounts of missing data.

### Reasons for non-vaccination

**Fig 2:**
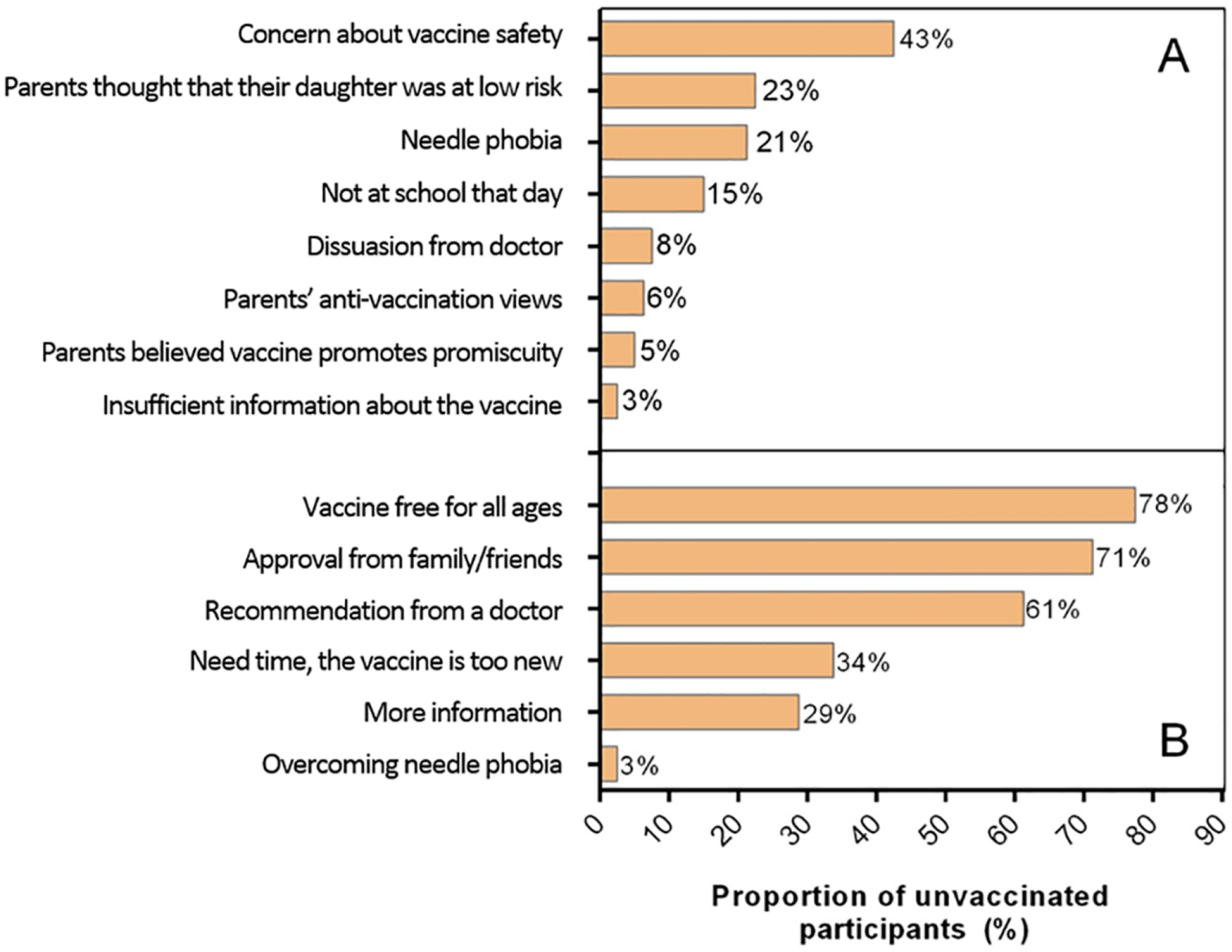
Reported reasons for not receiving the HPV vaccine (A) and; measures to improve HPV vaccination rates (B) among unvaccinated female participants who were eligible for the HPV vaccine between 2007 and 2009 as part of the National HPV Vaccination Program.

Unvaccinated participants were asked to report their reasons for why they believed they were not vaccinated. The most commonly reported reasons included parental concern about vaccine safety (42.5%), parental perception of their daughter being at low risk of HPV infection (22.5%) or having a needle phobia (21.3%). Fifteen percent (15.0%) of unvaccinated participants reported practical barriers to HPV vaccination, including being absent at school and forgetting to bring their signed consent forms on the day of vaccination. Only 5.0% reported parental belief that HPV vaccination promoted promiscuity as the reason for not being vaccinated (Fig 2A). When stratified by age at program commencement, parental concern about vaccine safety was reported by 57.6% of women who were <18 years of age, compared to only 4.7% of those who were 18 years and over (p<0.001).

Unvaccinated participants were asked to report factors that would encourage them to get the HPV vaccine (Fig 2B). The most commonly reported factors to encourage HPV vaccination included that vaccination was free (77.5%), and receiving a recommendation by a doctor to get vaccinated (61.3%). Participants reported that they were more likely to accept HPV vaccination, if they believed their family and friends approved of their decision to become vaccinated (71.3%). Some participants believed the HPV vaccine to be still new, but might become more likely to accept the vaccine with time (33.8%).

## Discussion

In this study of 417 young women living in Victoria Australia, we investigated attitudes, knowledge and factors associated with receipt of the HPV vaccine in the National HPV Vaccination Program. Compared with the vaccinated, unvaccinated women were more likely to be smokers and were less likely to use contraception. Unvaccinated women were also more likely to report neutral or negative attitudes towards the HPV vaccine and vaccinations in general. Sexual behaviour and HPV knowledge did not differ between the two groups. Independent factors associated with receipt of the vaccine in the school-based program included being Australia born and having completed childhood vaccinations. Among unvaccinated women, parental concerns about vaccine safety and perceived low risk of HPV infection for their daughters were predominant reasons for non-vaccination. The results of the study emphasize the need for long-term monitoring of the knowledge, attitude and beliefs towards HPV vaccination in the community to ensure high uptake of the vaccine and success of the program.

In Australia, those offered HPV vaccination through the school-based program are provided with information on the vaccine at the same time of receiving the consent form [27]. Despite this, nearly half (43%) of unvaccinated participants in this study reported parental concerns about vaccine safety as the main reason for not receiving the vaccine. Published Australian research suggests that when presented with facts about the HPV vaccine, some parents remained hesitant towards HPV vaccination due to an underlying mistrust of the health care system and belief of vaccinations to cause disability [28]. In fact, unvaccinated women in our study were significantly more likely to report incomplete childhood vaccinations in general. These findings are consistent with published Australian and international data suggesting that HPV non-vaccination is partly driven by parental disapproval of vaccination in general [29, 30].

Studies on HPV vaccine acceptability have reported parents’ health behaviours, beliefs and knowledge about HPV are significant predictors of parental intent for vaccination [31, 32]. In a US study of linked electronic health records of girls (aged 9 to 17 years) the authors found that mothers’ attitudes about preventative measures such has the Pap test influenced their adolescent daughters update of the HPV vaccine [32]. In turn, individual attitudes and beliefs around health and vaccination are likely to be influenced by parental views and behaviours [33–35]. Unvaccinated participants in our study were more likely to be smokers, were less likely to use contraception and had incomplete attendance at cervical screening, although the latter did not reach significance. Unvaccinated participants were also more likely to report neutral or negative attitudes towards vaccinations (including the HPV vaccine), and report neutral or negative attitudes towards future intentions of vaccinating their own children, suggesting they themselves were hesitant towards vaccination.

Among unvaccinated women who reported neutral or negative attitudes towards vaccinations, the majority took a neutral position. Research into parental opinions on immunisation have identified a continuum of views on vaccine hesitancy ranging from unquestionable acceptor through hesitancy to refuser [36, 37]. Accurate understanding of an individual’s HPV perception is critically important and may help guide effective strategies to promote vaccine acceptance. This is important given the fact that those identified as having a lesser degree of hesitancy are more likely to accept full vaccination uptake [38].

Primary health care provider recommendation remains a key strategy to increase HPV vaccination rates and promote vaccine acceptance [39, 40]. In our study, GPs were the most trusted sources of HPV information, with 61% unvaccinated participants reporting that they would accept HPV vaccination if it were recommended by a GP. Health care providers (GPs or nurses within a practice) play an important role in providing information and services for women, and are well placed to encourage participation in cervical screening. While the HPV vaccine is no longer freely available outside of the target age-range, it is important that heath care providers continue to use opportunistic visits by young women to engage in general discussion around HPV prevention, including vaccination. Providing young women with the opportunity to voice their concerns should form a central part of these discussions. Evidence based frameworks have been developed to assist health professionals when communicating with parents about vaccination [37]. Such frameworks should be expanded to include young adults, regardless of where they are along the decision-making trajectory.

In line with previous literature, this study showed that being born overseas was associated with lower HPV vaccine uptake [16]. This is in part driven by families who have arrived in Australia outside of the catch-up program window. However, published research has demonstrated that coverage of various vaccinations are lower in new migrants to Australia. This was attributed to a number of factors including language barriers, lack of specialised migrant health services, lack of awareness in migrants of their rights within the Australian health system, and cultural differences in attitudes towards preventative health care [41]. Australia is ethnically diverse and in 2011, 27% of Australians were first generation migrants [42]. It is therefore essential for public health prevention programs to target new migrants using culturally sensitive approaches, to ensure equitable delivery of vaccination to all population subgroups.

This study has several limitations. First, the retrospective nature of the study design means that it may be subjected to recall bias. Furthermore, the views of parents were reported by the participants rather than the parents themselves and may therefore be inaccurate or misconstrued. Second, reasons for non-vaccination are likely to be different in the catch-up program than in the school program. However in our study, the proportion of women who were aged 18 years and over at the time of the catch-up program was small (23%). Therefore, we had limited power to investigate factors associated with HPV vaccination in this group. Third, we did not demonstrate an association between geographic remoteness and uptake of the HPV vaccine. While this is consistent with published Australian data which shows a relatively equal uptake across urban and regional areas, lower vaccine completion rates have been reported among residents of remote areas [43]. It is likely that our study lacked power to demonstrate this association as only 19 women resided in outer regional Australia and none resided in remote Australia at the time of the catch up program. Last, our sample was limited to Victorian women only. While recruitment via social media has been shown to yield a broadly representative sample when compared with age-equivalent census data [44], there are substantial geographical and population variations between states and territories [27, 45] therefore the results may not be generalizable to all Australian women. Furthermore, the results may not be generalizable to other countries due to differences in delivery models of HPV vaccination.

## Conclusions

In summary, attitudes towards health, HPV infection and vaccinations may impact on the success of the HPV vaccination program. It is important for public health campaigns to continue to emphasise the efficacy and safety of the HPV vaccinations as a preventative health measure. Furthermore, long-term monitoring of the knowledge, attitude and beliefs towards HPV vaccination in the community is critical to ensure a continued high uptake of the vaccine and success of the program into the future.

## Supporting Information

S1 File Tung data. (XLS)
